# Maternal Iron Deficiency Programs Offspring Cognition and Its Relationship with Gastrointestinal Microbiota and Metabolites

**DOI:** 10.3390/ijerph17176070

**Published:** 2020-08-20

**Authors:** Hsin-Yi Hsieh, Yu-Chieh Chen, Mei-Hsin Hsu, Hong-Ren Yu, Chung-Hao Su, You-Lin Tain, Li-Tung Huang, Jiunn-Ming Sheen

**Affiliations:** 1 Department of Pediatrics, Kaohsiung Chang Gung Memorial Hospital and Chang Gung University College of Medicine, Kaohsiung 833, Taiwan; sally3735@cgmh.org.tw (H.-Y.H.); gesicht27@cgmh.org.tw (Y.-C.C.); a03peggy@cgmh.org.tw (M.-H.H.); yuu2002@cgmh.org.tw (H.-R.Y.); tainyl@cgmh.org.tw (Y.-L.T.); 2 Department of Pediatrics, Chiayi Chang Gung Memorial Hospital and Chang Gung University College of Medicine, Chiayi 613, Taiwan; b9302049@cgmh.org.tw

**Keywords:** spatial deficit, iron deficiency, microbiota, programming

## Abstract

Iron is an essential micronutrient for the brain development of the fetus. Altered intestinal microbiota might affect behavior and cognition through the so-called microbiota-gut-brain axis. We used a Sprague-Dawley rat model of a maternal low-iron diet to explore the changes in cognition, dorsal hippocampal brain-derived neurotrophic factor (BDNF) and related pathways, gut microbiota, and related metabolites in adult male offspring. We established maternal iron-deficient rats by feeding them a low-iron diet (2.9 mg/kg), while the control rats were fed a standard diet (52.3 mg/kg). We used a Morris water maze test to assess spatial learning and long-term memory. Western blot (WB) assays and a quantitative reverse-transcription polymerase chain reaction (qRT-PCR) were used to detect the BDNF concentration and related signaling pathways. We collected fecal samples for microbiota profiling and measured the concentrations of plasma short-chain fatty acids. The adult male offspring of maternal rats fed low-iron diets before pregnancy, during pregnancy and throughout the lactation period had (1) spatial deficits, (2) a decreased BDNF mRNA expression and protein concentrations, accompanied by a decreased TrkB protein abundance, (3) a decreased plasma acetate concentration, and (4) an enrichment of the *Bacteroidaceae* genus *Bacteroides* and *Lachnospiraceae* genus *Marvinbryantia*. Maternal iron deficiency leads to an offspring spatial deficit and is associated with alternations in gastrointestinal microbiota and metabolites.

## 1. Introduction

Iron deficiency has become one of the prevalent nutritional problems in the developing and developed world [1], particularly in preschool-age children and reproductive-age women [2]. Iron concentration is the rate-limiting factor of erythropoiesis, which may be insufficient when the serum iron level is below 50 μg/dL [3]. Furthermore, during pregnancy, the iron demand for the expanding blood volume and growth of the fetus, placenta, and other maternal tissues were increased, therefore raising the risk of iron deficiency anemia (IDA), especially in those lacking sufficient iron supplementation during the middle and late trimesters of pregnancy [4].

The main manifestations of IDA are palpitations, pallor, anorexia, dyspnea, and dizziness. Maternal iron deficiency might adversely affect maternal and fetal health and become one of the risk factors for offspring intrauterine growth retardation and adulthood cardiovascular disease [5]. Moreover, children with a poor iron status in the uterus have markedly worse fine motor skills and language ability [6]. In animal studies, prenatal iron deficiency is associated with neurochemistry alternations and a reduced hippocampal size in adulthood [7]. Furthermore, despite an early postnatal iron supply, the hippocampal size and behavioral deficits persisted into adulthood [8].

Neurotrophic factors, or “growth factors”, are directly involved in neuronal and synaptic growth. A small dimeric protein, called brain-derived neurotrophic factor (BDNF), which is widely expressed in the mammalian brain, and is vital for cognitive performance, spatial learning, and memory [9], as well as critical for neuronal synaptic plasticity, neuronal survival, and memory processing. Both human and animal studies have shown that maternal iron deficiency affects the fetal production of BDNF and hippocampal morphogenesis [10,11]. Moreover, in many neurological and psychiatric diseases, such as Parkinson’s disease, Huntington’s disease, schizophrenia, Alzheimer’s disease, and depression, the concentration of BDNF was found to be low [12,13].

Recent studies have shown that through neural, immune, and endocrine pathways, gut microbiota might communicate with the central nervous system (CNS) and influence brain morphology, brain function and personal behavior [14]. Infants with iron deficiencies also showed gut microbiota dysbiosis [15]. Thus, we studied whether maternal iron deficiency affects offspring cognition and the changes in gut microbiota and related metabolites in the offspring.

## 2. Materials and Methods

### 2.1. Animals and Ethics

A total of 12 virgin Sprague-Dawley (SD) rats (6 weeks old) were purchased from BioLASCO Taiwan Co., Ltd. (Taipei, Taiwan). The animals were housed in the animal care facility under standard photoperiod conditions (12 h light/dark cycle with lights on at 7 am) and were provided ad libitum access to water and food throughout the study. The protocols described herein were conducted with the approval of the Animal Care and Use Committee (Chang Gung Memorial Hospital, Kaohsiung, Taiwan, No. 2018110201) and designed to minimize the suffering and numbers of animals during the experiments.

### 2.2. Diet and Experimental Protocols

All purified diets were based on the AIN-93G diet (Research Diets Inc, New Brunswick, NJ, USA). By adding ferric citrate, we obtain the following iron concentrations: 52.3 mg/kg in the control diet (D10012G) and 2.9 mg/kg in the low-iron diet (D03072501), which was published previously [16] (Supplementary Table S1). The other composition of purified diet was identical.

The rats were randomly assigned to the control diet or the low-iron diet. After 3–6 weeks of respective diets, blood from the tail vein was drawn to check the iron profile and hemoglobin concentrations. Next, the female SD rats and male rats were allowed to mate. After 24 h, they were separated and the female rats were housed in a standard plastic cage individually.

Iron-deficient rats were then randomly divided into three groups: (1) a control diet through pregnancy and lactation, (2) a low-iron diet during the pregnancy period but control diet during the lactation period, and (3) a continued low-iron diet through pregnancy and lactation. Only male rats were enrolled in this study to decrease gender interference. Therefore, we had four groups of adult male offspring: (Figure S1).

1. Four-month-old male offspring of mothers receiving the control diet (sham control (SC group)) (*n* = 5).
2. Four-month-old male offspring of mothers receiving the low-iron diet but the control diet through the pregnancy (ICC group) (*n* = 5).
3. Four-month-old male offspring of mothers receiving the low-iron diet during pregnancy but the control diet during lactation (IDC group) (*n* = 5).
4. Four-month-old male offspring from mothers continuously receiving the low-iron diet (IDD group) (*n* = 4).

After weaning, the offspring were separated from their mothers and fed the control diet. The Morris water maze testing was conducted when the rats reached 16 weeks of age. The offspring were sacrificed at 17 weeks of age by Rompun + Zoletil and exsanguination.

### 2.3. Behavioral Evaluation

To assess spatial learning and memory, the Morris water maze test was conducted 5–7 days before sacrifice [17]. The water maze was a circular tank 180 cm in diameter and 50 cm in height with uniform, nonreflective interior surfaces, filled with water to a depth of 25 cm at 26 ± 1 °C. A 12 cm-diameter-platform was submerged 1.5 cm below the water surface, hidden from the rats’ view. We marked the visual clues around the room in a constant location, and we set a video camera above the center of the pool, using a video-tracking system (Noldus, Ethovision, The Netherlands).

#### 2.3.1. Days 1–4: Spatial Acquisition Phase

On the first day, for habituate training environment, each rat was placed in the water pool for 60 s without a platform. From days 1–4, the rats were trained for six trials each day to locate and escape onto the platform. The starting point varied in a quasi-random order during each trial, and the distance between the starting point and platform was constant. The rats were left on the platform during the 30 s inter-trial interval. If a rat could not find the platform within 60 s, we would manually place it on the platform. We recorded the latencies (times from the start to reaching the platform), average swimming speed, and cumulative distance from the platform.

#### 2.3.2. Day 5: Probe Trial Phase

The platform was removed on day 5, and the rats received a single 60 s probe trial from a novel start position. To determine if the animal remembered the location of the platform, we measured the path and time spent in the quadrant where the platform was previously located. The probe trial was considered a measure of spatial reference memory [18].

### 2.4. Tissue Dissection and Collection

At 17 weeks of age, all animals were weighed, blood samples were collected by cardiocentesis, and euthanized under Rompun + Zoletil 1:1. The dorsal hippocampus was dissected. The plasma was collected, and the samples were stored at −80 °C until further analysis.

### 2.5. Quantitative Reverse-Transcription Polymerase Chain Reaction (qRT-PCR) Analysis

Briefly, we used TRI reagent (Sigma, St. Louis, MO, USA) to extract RNA from the dorsal hippocampus and removed the contaminating DNA via DNase I (Ambion, Austin, TX, USA). The RNA (2 μg) was reverse transcribed by using SuperScript II RNase H-Reverse Transcriptase with random primers (Invitrogen, San Diego, CA, USA). We omitted the enzyme to perform control reverse transcriptase reactions and amplified the PCR to ensure no DNA contamination. QuantiTect SYBR green PCR reagents (Qiagen, Valencia, CA, USA) were used to conduct a two-step qRT-PCR following the manufacturer’s protocol on a LightCycler 480 real-time PCR system (Roche Diagnostics Ltd., Taipei, Taiwan). The primers for BDNF and β-actin (as reference) were designed from rat-specific sequences. We ran all the samples in duplicate. For the relative quantification of the gene expression, the comparative threshold cycle (CT) method was employed. To obtain the ^Δ^CT, we subtracted the averaged CT from the corresponding averaged β-actin value for each sample. To obtain ^ΔΔ^CT, we subtracted the average control ^Δ^CT value from the averaged experimental ^Δ^CT. By calculating 2^−ΔΔCT^ for the experimental vs. control samples, we established the fold increase (Supplementary Table S2).

### 2.6. Western Blot (WB) Assay

The WB analysis was conducted as previously published [19]. We lysed total protein extracted from the dorsal hippocampal tissue in ice-cold radioimmunoprecipitation assay buffer with a protease inhibitor cocktail (Roche, Indianapolis, IN, USA) then centrifugation. We used a RC DC protein assay kit (Bio-Rad, Hercules, CA, USA) to determine the protein concentrations in the supernatants. The WB analysis was used to quantify the BDNF, TrkB, and CREB protein density. We used primary antibodies, including BDNF (1:1000, Santa Cruz, CA, USA), TrkB (1:1000, Cell Signaling, Denver, MA, USA), and CREB (1:100; Millipore, Billerica, MA, USA), followed by secondary antibodies. We used enhanced chemiluminescence reagents (PerkinElmer, Waltham, MA, USA) to visualize the protein–antisera complex. The complex was then subtracted of background and quantified by densitometry (Quantity One Analysis software; Bio-Rad, Hercules, CA, USA) as the integrated optical density (IOD). The IOD was normalized to Ponceau red staining as an internal control (Supplementary Table S3).

### 2.7. Measurement of Plasma Acetate, Butyrate, and Propionate Concentrations

The blood samples collected via cardiocentesis were stored at –80 °C before examination. The plasma acetate, propionate, and butyrate concentrations were measured by gas chromatography–mass spectrometry (GC-MS, Agilent 7890, Agilent: Santa Clara, CA, USA). For metabolite extraction, we transferred a 0.15 mL sample into 1.5 mL Eppendorf tubes, added 0.05 mL 50% H_2_SO_4_ and 0.2 mL 2-methylvaleric acid (25 mg/L stock in methyl tert-butyl ether) as an internal standard, mixed by vortexing for 30 s with oscillations for 10 min, and then treated the samples with ultrasound for 10 min (incubated in ice water). The samples were centrifuged at 10,000 rpm at 4 °C for 15 min, and then kept at −20 °C for 30 min. Finally, the supernatant was transferred into a fresh 2 mL glass vial for GC-MS analysis, using an Agilent 7890B gas chromatograph system with an Agilent 5977B (Agilent: Santa Clara, CA, USA) mass spectrometer. A HP-FFAP capillary column was used. We injected a 1 μL aliquot of the analyte in split mode (5:1). We used helium as the carrier gas with a flow rate of 1 mL/min through the column; the front inlet purge flow was 3 mL/min. We maintained the initial temperature at 80 °C for 1 min, then raised it 5 °C/min to 150 °C, and finally raised it at a rate of 40 °C/min to reach 230 °C which was maintained for 12 min. The energy was –70 eV in the electron impact mode. The injection, transfer line, quad, and ion source temperatures were 240 °C, 240 °C, 230 °C, and 150 °C, respectively. We acquired the mass spectrometry data in Scan/SIM mode with the *m/z* range of 33–200 after a solvent delay of 5 min.

### 2.8. Gut Microbiota Profiling

We extracted bacterial DNA from frozen fecal samples following the manufacturer’s instructions and then amplified it by forward primers (5′-CGTCGGCAGCGTCAGATGTGTATAAGAGACAGCCTACGGGNGGCWGCAG-3′) and reverse primers (5′-GTCTCGTGGGCTCGGAGATGTGTATAAGAGACAGGACTACHVGGGTATCTAATCC-3′), to target the V3–4 region of the 16S rRNA gene. We analyzed the next-generation sequencing data with the Microbial Genomics Module of CLC Genomics Workbench 9.5.4 (Qiagen, Stockach, Germany). We used the Illumina MiSeq platform (Illumina, San Diego, CA, USA) and 16S Amplicon Sequencing (Illumina, San Diego, CA, USA) to sequence and amplify the RNA with a 600-cycle sequencing reagent. In order to identify the microbial marker, an analysis (linear discriminant analysis (LDA)) effect size was used. The LDA score indicates the differences in the genus–concentration abundance between the grouping categories.

### 2.9. Statistical Analyses

The results of biochemical parameters, the WB and qRT-PCR, were analyzed by an independent Student’s t test or one-way analysis of variance (ANOVA) with the least significant difference (LSD) post-hoc test. The average of the sum of the six trials per day of the spatial acquisition test was analyzed by an ANOVA and repeated-measures ANOVA; the day and groupwere considered independent variables. The taxonomic relative abundance profiles were compared using a Student’s *t*-test. All analyses were performed using SPSS (version 22.0, IBM Corp, Armonk, NY, USA). The significance was defined as *p* < 0.05, and values are expressed as the mean ± the standard error of the mean for all tests.

## 3. Results

### 3.1. Maternal Body Weight and Serum Iron and Hemoglobin Concentrations

**Figure 1:**
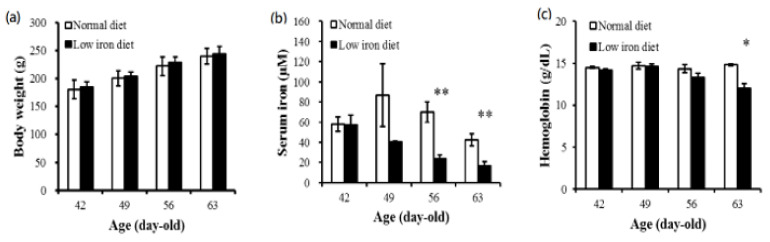
Body weights (**a**) and serum iron (**b**) and hemoglobin concentrations (**c**) of mother rats under normal and low-iron diets. Serum iron and hemoglobin were decreased from 56 days old and 63 days old separately. An independent Student’s t-test was used to assess the statistical significance of the differences between groups (*n* = 6). * *p* < 0.05 ** *p* < 0.01.

The body weight of maternal rats from 42 to 63 days old showed no significant difference between the low-iron diet group and the control diet group (Figure 1a). The concentration of serum iron in the mother rats was significantly lower after 14 days of consuming a low-iron diet compared to the rats in the control diet group (Figure 1b). Moreover, the hemoglobin concentration was significantly low after 3 weeks of the low-iron diet (Figure 1c).

### 3.2. Adult Male Offspring Body Weights and Serum Iron and Hemoglobin Concentrations

**Figure 2:**
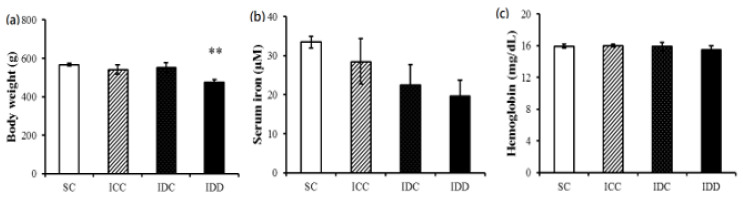
Body weights (**a**) and serum iron (**b**) and hemoglobin concentrations (**c**) of the offspring at age 16 weeks in four groups. A one-way analysis of variance (ANOVA) with a least significant difference correction was used to assess the statistical significance of the differences among groups, SC: Sham control, offspring from maternal control diet; ICC: offspring from a maternal low-iron diet but control diet during pregnancy; IDC: offspring from a maternal low-iron diet but control diet during lactation; IDD: offspring from a maternal low-iron diet through the pregnancy and lactation. ** *p* < 0.05 vs. SC.

At 16 weeks of age, we found that the body weights of the male offspring in the IDD group were lower than the rats in the SC group (Figure 2a). The serum iron concentration showed no significant differences among all four groups (SC, ICC, IDC, and IDD groups), although there was a decreasing trend from the SC group to the ICC, IDC, and IDD groups (Figure 2b). There was no marked difference in the hemoglobin concentrations among the four groups (Figure 2c).

### 3.3. Morris Water Maze

**Figure 3:**
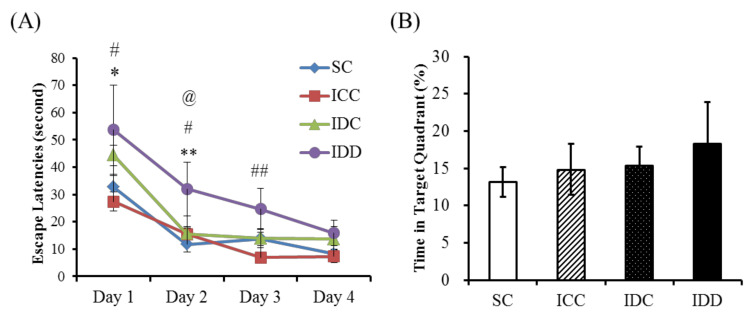
Morris water maze test. Latencies of escape to the platform (**A**) and time in the target quadrant (**B**). A two-way analysis of variance (ANOVA) with repeated measures (days) was used to assess the statistical significance of differences among the four experimental groups, * *p* < 0.05 vs. SC; ** *p* < 0.01 vs. SC; ^#^ *p* < 0.05 vs. ICC; ^##^ *p* < 0.01 vs. ICC; ^@^ *p* < 0.05 vs. IDC. Across 4 days of training, all mean escape latencies in each group decreased over the spatial acquisition phase. The ANOVA results show a significant reduction in the escape latency with training. The IDD group need more time finding the submerged platform than the other three groups. A probe trial administered on training day 5 showed no significant differences.

Figure 3 shows the mean escape latencies (A) and the percentage of time spent in the target quadrant (B). The swimming speed had no marked difference between the rats from each group and showed no mobility impairment (*p* > 0.1). It also demonstrated an improvement in the mean escape latencies during the 4 days of the spatial acquisition phase. However, there was a significant group difference. Rats in the IDD group needed more time to find the submerged platform than rats in the IDC (*p* < 0.05), ICC (*p* < 0.01), and SC (*p* < 0.01) groups. In the probe trial phase, there was no statistical difference in the time spent in the target quadrant among the four experimental groups (*p* > 0.05). The above findings indicated that iron deficiency caused an impairment in the acquisition phase of spatial learning and memory, but the spatial reference memory was not affected.

### 3.4. WB and qRT-PCR

**Figure 4:**
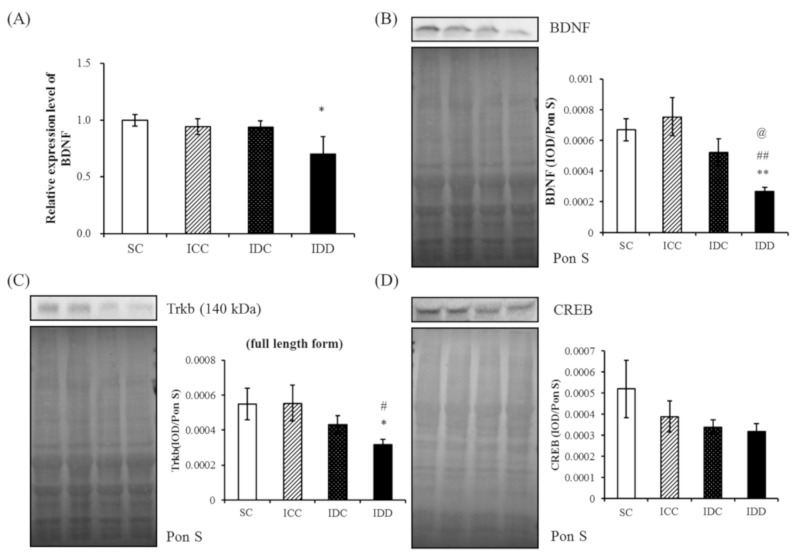
Brain-derived neurotrophic factor (BDNF) and related proteins in the dorsal hippocampus. Dorsal hippocampal *BDNF* mRNA expression (**A**), BDNF protein (**B**), TrkB protein (**C**) and CREB protein (**D**) in the four groups. BDNF mRNA, BDNF proteins, and TrkB proteins were all significantly lower in the IDD group. A one-way analysis of variance (ANOVA) with a least significant difference post-hoc test was used to assess the statistical significance of the differences among groups, * *p* < 0.05 vs. SC; ** *p* < 0.01 vs. SC; ^#^ *p* < 0.05 vs. ICC; ^##^ *p* < 0.01 vs. ICC; ^@^ *p* < 0.05 vs. IDC.

We next examined whether an iron deficiency altered the mRNA and protein concentrations of the BDNF signaling pathway within the dorsal hippocampus. The BDNF mRNA concentration was lower in the IDD group than in the SC group (*p* < 0.05) (Figure 4A). Moreover, BDNF protein abundance was significantly lower in the IDD group than the other three groups (*p* < 0.01) (Figure 4B). The full-length form of TrkB was lower in the IDD group than the SC and ICC groups (*p* < 0.05) (Figure 4C). There was no marked difference in CREB protein abundance among the four groups (Figure 4D). Our results imply that maternal iron deficiency may program adult male offspring, reducing the dorsal hippocampal BDNF concentration and altering the TrkB cascade, which is required for spatial learning and memory.

### 3.5. Short-Chain Fatty Acids (SCFAs)

**Figure 5:**
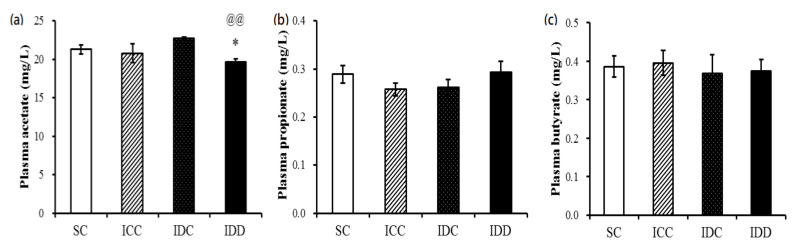
Plasma short-chain fatty acid concentrations. Acetate (**a**), propionate (**b**), and butyrate (**c**) concentration. A one-way analysis of variance (ANOVA) with a least significant difference post-hoc test was used to assess the statistical significance of the differences among groups, * *p* < 0.05 vs. SC; ^@@^ *p* < 0.01 vs. IDC.

Volatile fatty acids, also known as SCFAs, have been linked to the relationship among diet, gut microbiota, and host energy metabolism. In this study, the concentrations of acetate were marked lower in the IDD group than the other three groups. There was no significant difference in the concentrations of propionate and butyrate among the four groups (Figure 5).

### 3.6. Gut Microbiota

**Figure 6:**
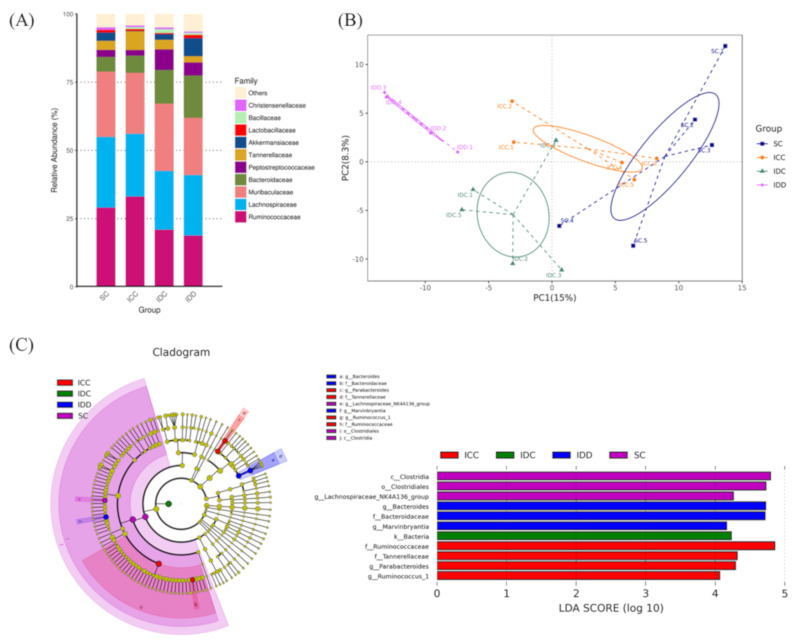
Gut microbiota of the offspring at 16 weeks of age in four groups. Relative abundances of the gut microbiota at the family concentration (**A**), principal component analysis (**B**), cladogram generated from the linear discriminant analysis (LDA) and LDA effect size to identify the enriched bacterial species (**C**).

16S rRNA sequencing was used to evaluate the richness, composition, and diversity of the gut microbiota. The four most abundant taxa were *Ruminococcaceae*, *Lachnospiraceae*, *Muribaculaceae*, and *Bacteroidaceae*. There was a greater enrichment of *Bacteroidaceae* in the IDD and IDC groups than in the SC and ICC groups (Figure 6A). A principal component analysis for beta diversity showed a clear segregation of all four groups (IDD, IDC, ICC, and SC) (Figure 6B). We found three differentially abundant taxonomic classes in the IDD group using an LDA effect size calculation with an LDA score higher than 4.0. The results showed that the *Bacteroidaceae* genus *Bacteroides* and *Lachnospiraceae* genus *Marvinbryantia* were significantly increased in rats in the IDD group compared to rats in the other groups (Figure 6C).

## 4. Discussion

In this study, we showed that maternal iron deficiency may program and alter adult male offspring development with regard to spatial learning and memory, dorsal hippocampus BDNF expression, gut microbiota, and SCFA concentrations. Our results showed that the adult male offspring of rats that were fed a low-iron diet before pregnancy and throughout the lactation period had (1) spatial deficits via a Morris water maze evaluation; (2) decreased dorsal hippocampal BDNF mRNA and protein concentrations accompanied by a low TrkB abundance; (3) a decreased plasma acetate concentration without changes in butyrate and propionate concentrations; (4) enrichment of the *Bacteroidaceae* genus *Bacteroides* and *Lachnospiraceae* genus *Marvinbryantia*.

It has long been known that iron is essential in the regulation of neuronal metabolism, especially during rapid neurologic development [20,21]. In both animal and human studies, perinatal iron deficiency has been shown to have detrimental programming effects and might cause long-lasting abnormalities in the learning and memory behavior of offspring [22,23], and these effects persisted even when the iron deficiency was corrected [24]. Our results revealed that the offspring of rats that underwent iron restriction before conception and throughout gestation and lactation displayed the poorest performances in the Morris water maze, with a corresponding decrease in the dorsal hippocampus BDNF concentration even after the iron and hemoglobin concentrations were restored. There were no significant differences in performance in the water maze test, or the BDNF and acetate concentrations when comparing the IDC, ICC and SC groups. Whether the iron correction during lactation had benefits in terms of preventing spatial memory impairments needs further investigation. Mihaila et al. stated there was no neural impairment if the maternal iron deficiency was initiated from the third trimester of pregnancy [25]. Additionally, Ranade et al. reported that, in the pups of rats that had insufficient iron throughout pregnancy, lactation suffered from the complete spectrum of defects compared to those from rats that had insufficient iron only during the pregnancy [26], indicating that the cognitive effects was correlated with the severity and duration of iron restriction. Moreover, iron deficiency without anemia also had a neurological effect suggesting that iron statuses should be checked early instead of only after the hemoglobin concentration changed.

### 4.1. BDNF Pathway

BDNF is an essential growth factor for the regulation of neuronal differentiation and synaptic plasticity that also plays a role in pre- and postnatal brain development [27,28]. The highest BDNF concentrations in the CNS were found in the hippocampus, frontal cortex, and amygdala [29]. Both serum BDNF concentrations and hippocampal volumes of human neonates born to iron-deficient mothers are significantly low, and the magnitude of reduction is proportional to the severity and duration of the maternal iron deficiency [11]. TrkB is a downstream signaling cascade that results in neuronal survival, differentiation, and synaptic plasticity. Phosphorylated TrkB activated by BDNF follows the general scheme for tyrosine kinases receptor and initiates signaling casacades. TrkB then enters into the nucleus and activates CREB to regulate gene expression for the differentiation and survival of neurons [30,31]. In this study, BDNF and TrkB were significantly low in the IDD group, although the decrease in CREB abundance was not significant. Our findings suggest that fetal and perinatal iron homeostasis is important for the expression of BDNF in brain development, and provides a molecular basis for the spatial learning deficits related to maternal iron deficiency.

### 4.2. Neuronal and Metabolic Pathways between the Brain and Intestine

Although the mechanistic pathway is not yet clear, the intestine and brain communicate by neuronal, immune and metabolic pathways [32]. In the neuronal pathway, the hippocampal BDNF concentration is decreased by antibiotic-induced gut dysbiosis, which could cause cognitive impairment [33]. SCFAs, including acetate, butyrate, and propionate, are metabolic substances produced by the gut microbiome. These substances also have anti-inflammatory functions that reduce cytokine concentrations, thus, increasing BDNF concentrations [34]. Decreased concentrations of SCFAs are associated with the alteration of microorganisms and a reduced secretion of BDNF [35]. Kobayashi et al. reported that the administration of acetate could improve memory function and behavior impairment [36]. Our studies showed that the acetate concentration decreased in the IDD group compared to that of the other three groups, which was consistent with the findings of Kobayashi et al.

### 4.3. IDA and Microbiota

Increasing evidence has revealed the importance of the gut–brain axis and has suggested that intestinal microbiota dysbiosis might lead to the dysregulation of the CNS and cause CNS impairment [37,38]. The diversity and composition of microbiota are controlled by host genes and environmental factors, such us diet, medication, and stress [39]. Additionally, changes in iron availability to the gut microbiota might alter the composition of siderophilic organisms, such as *Escherichia coli* and *Salmonella*, resulting in associated diseases [40]. In this study, a clear segregation of bacterial beta diversity was observed. Although there was no anemia during the examination, the gut microbiota diversity and composition were still different, illustrating that early iron restriction may have a long-lasting effect on gut microbiota. Our results are consistent with those of studies in which the iron-restricted group had a high composition of *Bacteroidaceae* and *Lachnospiraceae* [41]. Saji et al. reported that the fecal micriobiome, such us *Bacteroides*, was found to have a higher prevalence in cognitive impairment patients [42]. Arciniegas et al. stated that traumatic brain injury induced congitive deficits, and Treangen et al. found more abundant *Lachnospiraceae* in microbiota after a traumatic brain injury [43,44]. However, we could not completely explain how the change in gut microbiota affects the plasma acetate concentration, indicating that some uncovered mechanism must be involved. The association between the microbiota and SCFA alteration in rats with IDA requires further investigation.

There were several limitations to our study. We only studied male offspring to reduce the gender difference, and there were only four rats in the IDD group. Therefore, we believe that future studies must include experiments with more number of rats and female offspring.

## 5. Conclusions

It has been suggested that maternal iron deficiency is associated with offspring cognition impairment, decreased BDNF concentrations, low plasma acetate concentrations, and an altered microbiota. Therefore, improving the nutritional status of pregnant women could have a positive effect on the future brain development of their offspring. Further studies are required to verify whether treatment with probiotic, nonviable components of a bacterium, or its metabolite could alter the microbiota and ameliorate cognitive dysfunction.

## Supplementary Materials

The following are available online at https://www.mdpi.com/1660-4601/17/17/6070/s1, Table S1: Composition of diets, Figure S1: Animal grouping, Table S2: qRT-PCR primer sequences, Table S3: Western blot antibody.
