# Supplementary material for: Maternal Iron Deficiency Programs Offspring Cognition and Its Relationship with Gastrointestinal Microbiota and Metabolites
Source: Int J Environ Res Public Health. 2020 Aug 20;17(17):6070. doi: 10.3390/ijerph17176070 (PMC7504367; doi:10.3390/ijerph17176070)
Supplement: Supplementary file 1 [file ijerph-17-06070-s001.pdf]

Table S1. Composition of diets.

	Control Diet		Low-Iron Diet	
	Concentration (gm)	mg Fe added (calculated)	Concentration (gm)	mg Fe added (calculated)
Casein	200	1.7	200	1.7
L-Cystine	3	0.0048	3	0.0048
Corn starch	397.486	0.397486	397.486	0.397486
Maltodextrin 10	132	0.1056	132	0.1056
Sucrose	100	0.08	100	0.08
Cellulose, BW200	50	5	0	0
Avucel PH101	0	0	50	0.015
Soybean oil	70	0	70	0
t-Butylhydroquinone	0.014	0.00014	0.014	0.00014
Mineral Mix S10022G	35	45	0	0
Mineral Mix S18706	0	0	35	0.56
Ferric Citrate	0	0	0	0
Vitamin Mix V10037	10	0.0009	10	0.0009
Choline Bitartrate	2.5	0.0005	2.5	0.0005

Concentrations are expressed in gm.

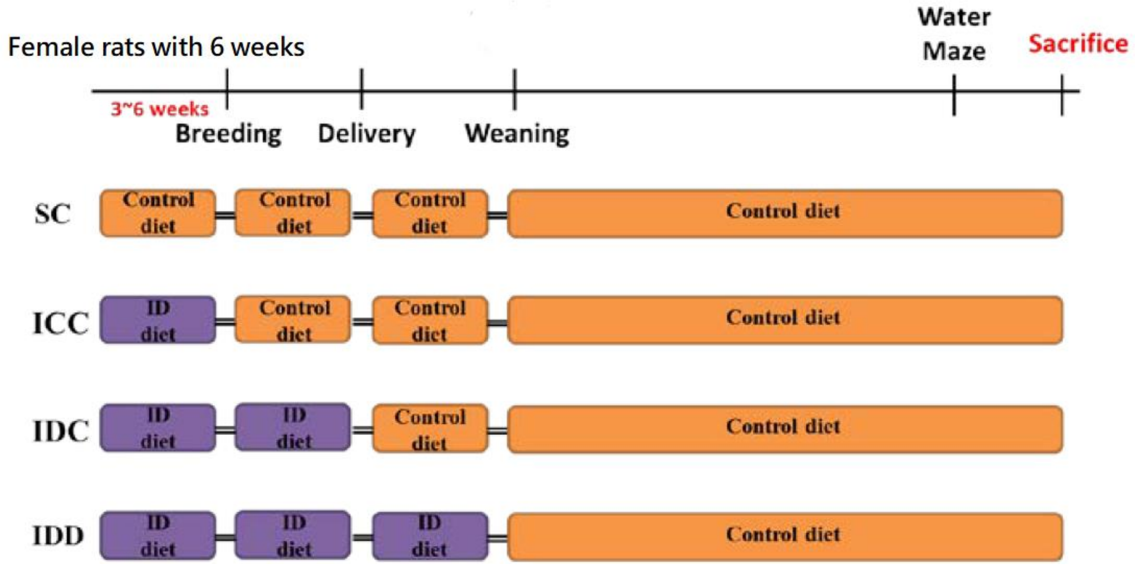

Figure S1. Animal grouping.

Table S2. qRT-PCR primer sequences.

Target Genes	Primer Sequences
BDNF	Forward: 5′ AGCTGAGCGTGTGTGACAGTAT 3′
	Reverse: 5′ CCGAACATACGATTGGGTAGTT3′
TrKB	Forward: 5′GCACATCGCTCAGCAAATCG 3′
	Reverse: 5′ACAACTCCCAGGCTCCAGAC 3′
CREB	Forward: 5′CCCAGGGAGGAGCAATACAG 3′
	Reverse: 5′GGGAGGACGCCATAACAACT 3′

β-actin	Forward: 5′TACTGCCCTGGCTCCTA-3′
	Reverse: 5′GGGCCGGACTCATCGTA-3′

Table S3. Western blot antibody.

Type	Antibody	Host	Dilution	Supplier
Primary/polyclonal	BDNF	rabbit	1:1000	Santa Cruz (CA, USA)
Primary/monoclonal	Trkb	rabbit	1:1000	cell signaling (Denver, MA, USA)
Primary/polyclonal	CREB	rabbit	1:1000	Millipore (Billerica, MA, USA)
Secondary/polyclonal	Anti-rabbit IgG (H+L)	Goat	1:10000	LEADGENE Biomedical (Taiwan)
